# Calcium/NFAT signalling promotes early nephrogenesis

**DOI:** 10.1016/j.ydbio.2011.01.033

**Published:** 2011-04-15

**Authors:** S.F. Burn, A. Webb, R.L. Berry, J.A. Davies, A. Ferrer-Vaquer, A.K. Hadjantonakis, N.D. Hastie, P. Hohenstein

**Affiliations:** a MRC Human Genetics Unit, Institute of Genetics & Molecular Medicine, Western General Hospital, Crewe Road, Edinburgh, EH4 2XU, UK; b Centre for Integrative Physiology, University of Edinburgh, George Square, Edinburgh, EH8 9XD, UK; c Developmental Biology Program, Sloan-Kettering Institute, New York, NY 10065, USA

**Keywords:** Kidney, Nephrogenesis, Wnt4, NFAT, Calcium, Embryo

## Abstract

A number of *Wnt* genes are expressed during, and are known to be essential for, early kidney development. It is typically assumed that their products will act through the canonical β-catenin signalling pathway. We have found evidence that suggests canonical Wnt signalling is not active in the early nephrogenic metanephric mesenchyme, but instead provide expressional and functional evidence that implicates the non-canonical Calcium/NFAT Wnt signalling pathway in nephrogenesis. Members of the NFAT (Nuclear Factor Activated in T cells) transcription factor gene family are expressed throughout murine kidney morphogenesis and NFATc3 is localised to the developing nephrons. Treatment of kidney rudiments with Cyclosporin A (CSA), an inhibitor of Calcium/NFAT signalling, decreases nephron formation — a phenotype similar to that in *Wnt4*^−/−^ embryos. Treatment of *Wnt4*^−/−^ kidneys with Ionomycin, an activator of the pathway, partially rescues the phenotype. We propose that the non-canonical Calcium/NFAT Wnt signalling pathway plays an important role in early mammalian renal development and is required for complete MET during nephrogenesis, potentially acting downstream of Wnt4.

## Research Highlights

► No β-catenin activity can be detected in the early nephrogenic mesenchyme. ► Expressional and functional data implicate Ca^2+^/NFAT Wnt signalling in nephrogenesis. ► Disruption of the Ca^2+^/NFAT pathway disrupts kidney morphogenesis. ► Increased Ca^2+^ signalling partially rescues nephrogenesis in *Wnt4* knockout mice. ► We propose Ca^2+^/NFAT signalling as a novel regulator of nephrogenesis.

## Introduction

The mammalian kidney develops as the product of inductive interactions between the ureteric bud (UB) and the adjacent metanephric mesenchyme (MM). The UB responds to cues from the MM to invade it at E10.5 (embryonic day 10.5) and from ~ E11.0 the bud undergoes branching morphogenesis. Concomitant with this branching the UB signals to the surrounding MM to commence nephrogenic differentiation. The first stage in nephrogenesis is the condensation of patches of induced MM to form renal aggregates. These aggregates subsequently undergo a mesenchymal to epithelial transition (MET), adopting a number of sequential forms: firstly epithelised renal vesicles, then comma-shaped bodies and subsequently s-shaped bodies, and finally mature nephrons — the functional filtration and solute recovery unit of the adult kidney.

Wnt signalling is an essential component of embryonic development and is required for a number of important morphogenetic processes during kidney development (Bridgewater et al., 2008; Carroll et al., 2005; Majumdar et al., 2003; Stark et al., 1994). Correct development of the embryonic kidney depends on a number of *Wnt* genes, including *Wnt2b*, *Wnt4*, *Wnt9b*, and *Wnt11* (Merkel et al., 2007). *Wnt4* is expressed in the condensing MM where it is required for MET. The renal aggregates, vesicles, comma-shaped bodies, s-shaped bodies, and nephrons which sequentially develop from this MM also express *Wnt4*. Mice without functional Wnt4 die shortly after birth due to renal failure (Stark et al., 1994). Ectopic introduction of Wnt4 protein into MM cultures can induce nephron formation in the absence of the UB (Kispert et al., 1998). These findings show Wnt4 is both necessary and sufficient for nephron induction, but which pathway it acts through is unclear.

In recent years a body of evidence has accumulated to suggest that the canonical β-catenin Wnt signalling pathway is insufficient for full nephrogenic MET. The pathway has been shown by a number of groups to be required for the initiation and maintenance of nephrogenesis (Kuure et al., 2007; Park et al., 2007; Schmidt-Ott et al., 2007). However, terminal MET is now thought to require attenuation of β-catenin signalling and the activity of Wnt4 through a second, non-canonical pathway (Lyons et al., 2009; Park et al., 2007). The best characterised non-canonical Wnt signalling pathways are the planar cell polarity (PCP) and Calcium/NFAT pathways. The PCP pathway has recently been shown to play a role in renal tubule morphogenesis, downstream of Wnt9b (Karner et al., 2009). The Calcium/NFAT pathway is activated upon Wnt-induced increase of intracellular Ca^2+^ (Saneyoshi et al., 2002). This rise in Ca^2+^ levels activates the phosphatase Calcineurin which dephosphorylates the NFAT transcription factors, facilitating their translocation to the nucleus and subsequent transcriptional activation of target genes. Five mammalian NFAT transcription factors have been identified, four of which are Calcineurin-regulated (NFATc1, NFATc2, NFATc3, and NFATc4) and one of which is not (NFAT5). Calcineurin/NFAT signalling regulates a range of cellular processes and plays essential roles during embryonic development of the heart, skull, and brain (Arron et al., 2006; Horsley and Pavlath, 2002).

A number of pieces of evidence point to a role for the Calcium/NFAT pathway in kidney development. Firstly, treatment of rabbit embryos *in utero* with Cyclosporin A (CSA), a Calcineurin inhibitor, reduces nephron number at birth (Tendron et al., 2003). CSA also inhibits growth of mouse embryonic kidneys in culture, resulting in decreased proliferation (Alcalay et al., 2007). Treatment of human proximal tubule cells with CSA results in apoptosis and causes renal fibrosis due to an epithelial to mesenchymal transition (EMT) (Slattery et al., 2005). In *Xenopus* CSA treatment also produces kidney defects (Yoshida et al., 2004), whilst increasing intracellular Ca^2+^ promotes pronephric tubule differentiation (Leclerc et al., 2008). In transgenic mice, loss of the Calcineurin A-α subunit results in a reduced nephrogenic zone, kidney agenesis, and postnatal lethality (Gooch et al., 2004). Furthermore, Polycystin-1 − the product of the *PKD1* gene mutated in ~ 85% of individuals with autosomal dominant polycystic kidney disease − activates the Calcium/NFAT pathway, components of which can be detected in late gestation and adult kidney (Puri et al., 2004). It has also been recently shown that NFATc1 may play roles in UB branching and glomerular development (Yi et al., 2010), and that conditional activation of *NFATc1* in developing podocytes causes glomerulosclerosis (Wang et al., 2010). Finally, *NFATc4* expression was observed in the embryonic kidney stroma and interstitium, following detection in a microarray screen of dynamically expressed renal development genes (Challen et al., 2005). On the basis of these findings, we investigated the potential role of Calcium/NFAT signalling during early kidney morphogenesis. We here present expressional and functional data to support a role for Calcium/NFAT signalling in nephrogenesis, possibly acting downstream of Wnt4.

## Results

### The β-catenin Wnt pathway cannot be detected in early nephrogenic tissue

**Supplementary Fig. 1:**
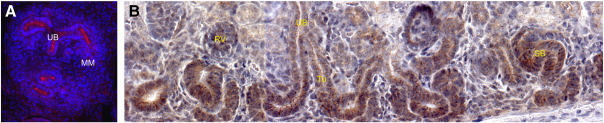
Pan β-catenin antibody staining. A) In E12.5 sectioned kidneys anti-pan-β-catenin antibody fails to detect β-catenin (red) in the condensed MM whilst strong localisation was found in the UB (DAPI-stained nuclei are blue). B) By E18.5 anti-pan-β-catenin localises to epithelial structures including the tubules and those derived from the MM: the renal vesicles, and comma- and s-shaped bodies. Protein can also be seen in the cap mesenchyme at this stage. MM — metanephric mesenchyme; RV — renal vesicle; SB — s-shaped body; Tu — tubule; UB — ureteric bud.

**Fig. 1:**
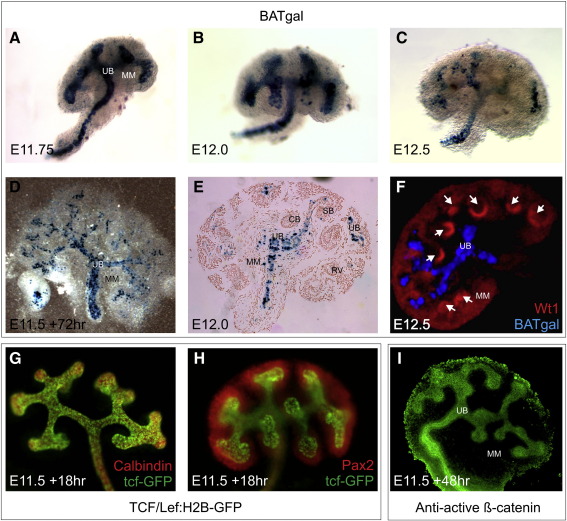
β-catenin signalling is not detectable in early nephrogenic tissues, suggesting that Wnt4 acts through a non-canonical pathway during nephrogenesis. A-F) X-gal staining in the BAT-gal reporter mouse confirms β-catenin signalling activity in the UB and absence of detectable activity in the MM and its derivatives, in A–C) ~ E11.75–E12.5 whole kidneys (not shown to scale), D) cultured kidney rudiments (E11.5 + 72 h), and E) sectioned E13.5 kidneys (counterstained with Nuclear Fast Red). F) Optical Projection Tomography (OPT) analysis of BAT-gal staining and immunolocalisation of Wt1 in an E12.5 kidney (condensed cap MM is denoted by white arrows). G–H) Direct fluorescence (green) from the product of the TCF/Lef:H2B-GFP reporter transgene in cultured (E11.5 + 18 h) kidneys with antibodies against G) Calbindin (red), a UB marker, and H) Pax2 (red), a marker of the MM. I) Immunohistochemistry on cultured kidneys (E11.5 + 48 h) for dephosphorylated (active) β-catenin (green). The signal around the edge of the cultured tissue is auto-fluorescence. MM — metanephric mesenchyme; UB — ureteric bud.

To study the role of β-catenin in mediating Wnt4 signals in renal development we examined the canonical Wnt signalling pathway during early nephrogenesis and found that, whilst highly active in the UB, it does not appear to be active in early nephrogenic tissues. We analysed β-catenin reporter activity in three transgenic mouse lines from E10.5 to 15.5. Reporter activity was absent from the condensing MM and nascent nephrons in the TOP-gal (data not shown), BAT-gal, and TCF/Lef:H2B-GFP lines. Strong BAT-gal activity was present in the UB but not in the surrounding condensed MM and developing nephrons where *Wnt4* is expressed in whole (Figs. 1A–C, F), cultured (Fig. 1D), and sectioned (Fig. 1E) embryonic kidneys. These data support the hypothesis that an alternative, non-canonical, Wnt pathway may be active in nephrogenesis. Similarly the TCF/Lef:H2B-GFP transgene is expressed only in the UB, and not in the condensing MM, during early nephrogenesis as shown by co-staining with antibodies against Calbindin, a UB marker (Fig. 1G), and Pax2, a marker of the MM (Fig. 1H). Furthermore, immunohistochemistry on E11.5 kidneys grown *ex vivo* for 48 h failed to detect active (dephosphorylated) β-catenin protein in the MM or condensing mesenchyme, whilst strong localisation was apparent in the UB (Fig. 1I). A pan-β-catenin antibody localised strongly to the UB but was also in evidence at lower levels in the MM at E12.5 (Supplementary Fig. 1A) and E18.5 (Supplementary Fig. 1B). The pan-β-catenin antibody also localised to the maturing nephrogenic structures at E18.5, reflecting the role of β-catenin in adherens junctions of epithelial tissue (Supplementary Fig. 1B). It should be noted that even in the UB – in which β-catenin signalling is known to be active – this pan β-catenin antibody was not nuclear localised, illustrating the current limits on investigating this pathway by immunohistochemistry and emphasising the importance of reporter models despite their possible limitations.

### Components of the Calcium/NFAT Wnt pathway are present in the developing kidney

**Fig. 2:**
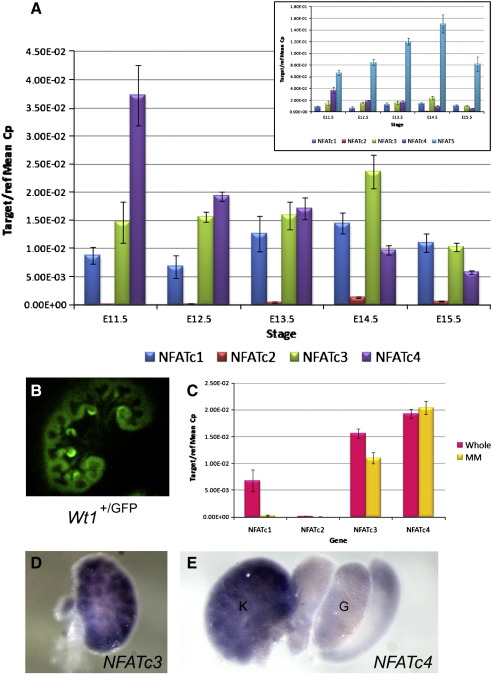
NFAT genes are expressed during kidney development. A) Quantitative real-time RT-PCR analysis, relative to GAPDH, of E11.5–15.5 whole kidneys reveals that all five NFAT genes are expressed. The main graph shows data on the calcium-regulated NFAT genes (*NFATc1-c4*); the inlay graph also includes data on the non-calcium-regulated *NFAT5* gene. B) Wt1-GFP (green) fluorescence in an embryonic kidney marking the MM and its derivatives. C) Expression analysis of NFATc genes in MM (Wt1-GFP positive) and whole kidney. Expression of D) *NFATc3* and E) *NFATc4* was confirmed by *in situ* hybridisation in E13.5 whole kidneys. G — gonad; K — kidney.

An activated mutant form of β-catenin has been shown to initiate nephron formation in the MM (Park et al., 2007). However, the absence of detectable endogenous β-catenin activity from the cap mesenchyme where Wnt4-induced MET occurs implies it cannot signal immediately downstream of Wnt4. To investigate the role of the non-canonical Calcium/NFAT Wnt signalling pathway in kidney development, we analysed expression of the NFAT transcription factors in embryonic kidneys using quantitative real-time RT-PCR (Taqman). All five NFAT genes were found to be expressed from E11.5–15.5 (Fig. 2A). The most highly expressed gene was *NFAT5*, whose product is not calcium-regulated but loss of which is known to produce a renal phenotype (Lopez-Rodriguez et al., 2004). Of the genes encoding calcium-regulated (NFATc) proteins, *NFATc4* exhibits the highest expression during early kidney development (E11.5–13.5), whilst *NFATc3* and *NFATc1* dominate at E14.5 and E15.5 respectively. Two distinct RT-PCR expression signatures were identified: 1) expression generally increasing from E11.5, peaking at E14.5 and then decreasing – as seen for *NFATc1*, *NFATc2*, *NFATc3*, and *NFAT5*; 2) expression highest at E11.5 then down regulated as renal morphogenesis progresses – as with *NFATc4*. We postulated that this second pattern may be associated with expression in the uninduced MM which is abundant at E11.5 then subsequently undergoes MET and differentiates into nephrons. To address this we isolated GFP-positive E12.5 MM from *Wt1*^+/GFP^ embryos (Hosen et al., 2007) using fluorescence-activated cell sorting (FACS) and repeated the Taqman analysis on cDNA prepared from this cell population. The *Wt1-GFP* knock-in transgene is expressed in the uninduced MM and subsequently the condensing MM, comma- and s-shaped bodies, and glomerular podocytes (Fig. 2B). We could therefore compare MM/MM-derived tissues (Wt1-GFP-positive) against whole kidney, thus confirming that *NFATc4* is the gene most enriched in the MM relative to the whole kidney (Fig. 2C). The other *NFAT* genes have higher relative expression in non-MM tissues at E12.5.

**Supplementary Fig. 2:**
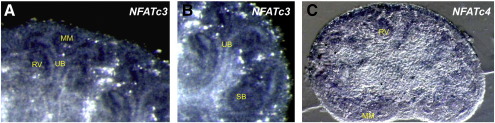
*NFATc3* and *NFATc4* expression. 70 μm vibratome sections of E13.5 kidneys previously stained whole mount for A–B) *NFATc3* and C) *NFATc4* expression by *in situ* hybridisation. MM — metanephric mesenchyme; RV — renal vesicle; SB — s-shaped body; UB — ureteric bud.

We next performed *in situ* hybridisation and confirmed renal expression of *NFATc3* (Fig. 2D) and *NFATc4* (Fig. 2E) at E13.5. *NFATc3* expression was detected in the UB, MM, and epithelial nephrogenic structures (Supplementary Fig. 2A–B). Expression of *NFATc4* was strongest in the MM (Supplementary Fig. 2C). Data from the GUDMAP renal expression database (http://www.gudmap.org), supports our findings: *NFATc3* expression is shown by *in situ* hybridisation in the E15.5 UB, MM, tubules, and renal interstitium (Andrew McMahon lab), and microarray data reveals *NFATc3* expression in the UB, MM, renal vesicles, s-shaped bodies, and podocytes at E11.5–15.5; microarray analysis of *NFATc4* expression also detected transcripts in the UB, renal vesicles, podocytes, and proximal tubules at E11.5–15.5, with very high expression in the E11.5 MM, confirming our RT-PCR data.

**Fig. 3:**
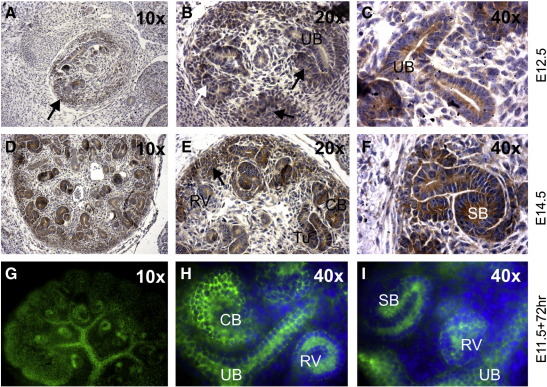
NFATc3 is present in the developing nephrons and UB. Immunoperoxidase antibody detection (brown) on sections of A–C) E12.5 and D–F) E14.5 kidneys, and G–I) fluorescent antibody detection (green) on E11.5 + 72 h cultured kidney rudiments. In (A–F) cell nuclei are stained blue with haematoxylin; in (G–I) nuclei are DAPI-stained blue. Higher magnification images are presented from separate kidneys to increase the range of data shown. CB — comma-shaped body; RV — renal vesicle; SB — s-shaped body; Tu — tubule; UB — ureteric bud. Black arrows: condensed MM.

A number of antibodies were tested to investigate more precisely the localisation of the NFAT transcription factors. We could not identify suitable antibodies against NFATc1 and NFATc4, but obtained data for NFATc3 and NFATc2. Immunohistochemistry on E12.5 sectioned kidneys (Fig. 3A–C), E14.5 sectioned kidneys (Fig. 3D–F), and E11.5 + 72 h cultured kidneys (Fig. 3G–I), revealed strong localisation of NFATc3 to both the condensing MM and epithelium (UB, renal vesicles, comma- and s-shaped bodies). This is in agreement with data in the Human Protein Atlas showing NFATc3 in adult renal tubules and glomeruli (http://www.proteinatlas.org; (Berglund et al., 2008)). NFATc2 was nuclear in the MM of E11.5 +72 h cultured kidney rudiments but appeared to be excluded from more mature aggregates (data not shown).

### Inhibition of calcium signalling disrupts renal morphogenesis

**Fig. 4:**
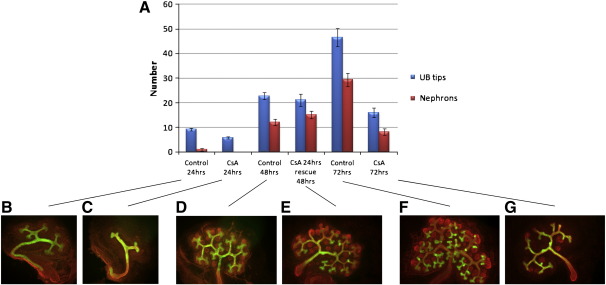
Inhibition of calcineurin/NFAT signalling by CSA disrupts renal morphogenesis. A) Cultured kidney rudiments were grown on control (0.1% ethanol) or CSA-supplemented (10 μM) media and the numbers of UB tips and nephrons (epithelised bodies within the MM, at all stages of nephrogenesis) counted. CSA treatment for 24 h (C) or 72 h (G) disrupted renal morphogenesis, when compared to ethanol-treated controls (B, F respectively). The number of both UB tips and nephrons was reduced. D, E) These phenotypes could be rescued by removing the CSA-supplemented media at 24 h and replacing it with control media for 48 h. (B–G) show immunohistochemistry on cultured kidneys using antibodies against Laminin (red; marks basement membrane of epithelia) and Calbindin (green; marker of UB epithelium).

Given that all the NFAT genes are expressed during renal morphogenesis and that developmental functional redundancy has been demonstrated between them in at least two organ systems (Arron et al., 2006), we decided to manipulate the entire Calcineurin/NFAT pathway pharmacologically to investigate its role in kidney development. CSA is an inhibitor of the pathway; when applied to cultured E11.5 CD1 kidney rudiments the number of UB tips and nephrons was reduced (Fig. 4A). The same effect was observed using FK506, a second Calcineurin/NFAT pathway inhibitor (data not shown), supporting the requirement for Calcineurin/NFAT signalling during renal development. Removal of CSA from the culture media rescued these phenotypes, indicating the observed phenotypes were not due to toxicity. Both drugs target Calcineurin itself and therefore distinguish between Ca^2+^ signalling through the NFAT/Calcineurin pathway and other Ca^2+^ pathway signals (Liu et al., 1991).

### Exogenous activation of calcium signalling disrupts renal morphogenesis

**Fig. 5:**
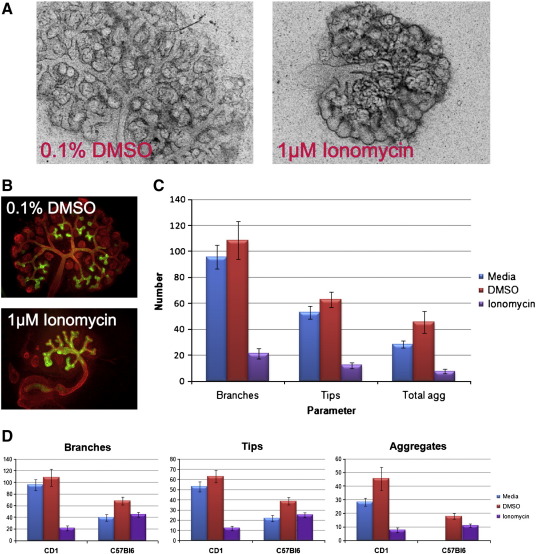
Ionomycin disrupts renal morphogenesis in a genetic background-dependent manner. A–B) Control and Ionomycin-treated CD1 kidneys after A) 5 or B) 3 days in culture. A) Bright field, B) immunofluorescence using antibodies against Calbindin (green) and Laminin (red). C) Numbers of branches, tips and aggregates in control and Ionomycin-treated cultured CD1 kidney rudiments. D) Comparison of branch, tip and aggregate numbers for control and Ionomycin-treated cultured CD1 and C57BL/6 kidney rudiments (data is not available for C57Bl6 aggregate number in non-supplemented media).

Exogenous activation of the Calcium/NFAT pathway by Ionomycin in E11.5 cultured kidneys disrupted renal morphogenesis. After five days in culture the UB became bloated (Fig. 5A). The UB branching pattern was also abnormal (Fig. 5B) and consequently the numbers of UB branches, tips, and renal aggregates were decreased compared to controls grown with Dimethyl sulphoxide (DMSO)- or non-supplemented media (Fig. 5C). The relative reduction was similar for all three parameters (branches, tips, and aggregates), and approximately the same ratios were maintained between parameters using all three types of media, demonstrating that the reduction in nephrogenesis was a result of decreased branching versus a nephron-specific effect. The morphology of the nephrons was normal. DMSO (the solvent for Ionomycin) on its own exerted a minor positive effect on all three parameters.

These findings were made using kidneys from wild type CD1 mice; intriguingly, when the experiments were repeated on the wild type C57Bl/6 background Ionomycin did not produce such a dramatic reduction in the number of branches, tips, or aggregates (Fig. 5D). DMSO however maintained the positive effect it had exerted on these parameters on the CD1 background, suggesting that specific buffering may exist in the C57Bl/6 strain to alterations in the Calcium/NFAT pathway. This was unexpected but noteworthy: genetic background should be borne in mind when designing and analysing these types of experiment.

### Ectopic activation of calcium signalling partially rescues the *Wnt4*^−/−^ kidney phenotype

**Fig. 6:**
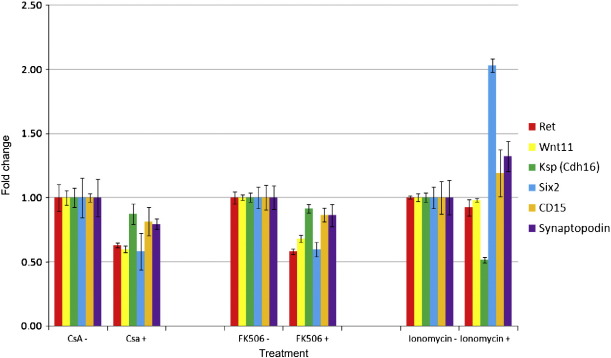
Disruption of calcium signalling reduces expression of UB genes but ectopic activation promotes nephrogenic gene expression. Quantitative real-time RT-PCR on E11.5 CD1 kidneys cultured for 72 h in the presence of CSA, FK506 or Ionomycin normalised to the relevant solvent-only control. CSA: 10 μM in ethanol; FK506: 10 μM in ethanol; Ionomycin: 1 μM in DMSO; ethanol: 0.1%; DMSO: 0.1%.

To further characterise the morphological defects present in CSA, FK506, and Ionomycin-treated kidney rudiments we next examined the differential expression of tissue-specific markers. E11.5 CD1 kidneys grown in the presence of each drug were investigated for expression of markers of the UB (*Ret*, *Wnt11*, *Ksp-cadherin* (*Cdh16*)), the cap MM (Six2), proximal tubules (*CD15*), and podocytes (*Synaptopodin*) (Fig. 6). CSA and FK506 showed highly comparable results as would be expected since both compounds act on Calcineurin. The UB markers were down regulated following either inhibition or ectopic activation of calcium signalling, confirming our earlier morphometric observations. Expression of the MM-associated genes was also decreased following inhibition — again, as expected. However, the MM-associated genes were up-regulated in Ionomycin-treated kidneys, suggesting a pro-nephrogenic role for calcium signalling. *Six2* exhibited the strongest up-regulation.

Induction of MM-associated genes by Ionomycin was unexpected given the decrease in the physical number of renal aggregates observed in Ionomycin-treated CD1 kidneys (Fig. 5C). However, the number of UB branches and tips is also greatly reduced in Ionomycin-treated kidneys; the reduction in aggregate number may simply therefore be a consequence of this phenotype as nephron number is dependent upon UB tip number. Thus the reduction in tip number could mask any pro-nephrogenic effect. To investigate this further we employed the *Wnt4*^−/−^ mouse, in which nephrogenesis is deficient. The *Wnt4* mutant was maintained on the C57Bl/6 background so adverse effects on wild type tissue would not be predicted, as discussed before (Fig. 5D).

**Fig. 7:**
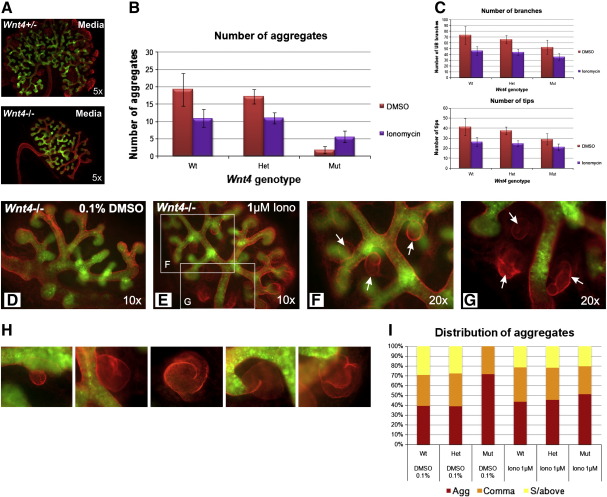
Ectopic activation of Calcium/NFAT signalling partially rescues the *Wnt4*^−/−^ phenotype. A) Nephrogenesis is disturbed in *Wnt4*^−/−^ kidneys, as demonstrated by the absence of Laminin (red) marked aggregates in an E11.5 kidney grown in non-supplemented media for 72 h (green: Calbindin). B) The total number of renal aggregates (aggregates, renal vesicles, comma shaped bodies, s-shaped bodies) in wild type, *Wnt4*^+/−^ and *Wnt4*^−/−^ E11.5 kidney rudiments after 72 h culture in control or Ionomycin-supplemented medium. C) Number of UB branches and tips in control and Ionomycin-treated cultures. D–G) Immunofluorescense of control (D) or Ionomycin-treated (E–G) *Wnt4*-deficient kidney rudiments from the same embryo using Laminin (red, epithelialised nephrons) and Calbindin (green, UB) antibodies. H) Enlarged examples of the different stages of nephron formation found in Ionomycin-treated kidneys (not to scale). I) Quantification of different stages of renal aggregates.

The renal phenotype generated following CSA/FK506 inhibition of Calcineurin/NFAT signalling is reminiscent of that in the *Wnt4*^−/−^ mouse (a reduction in the number of nephrons) and supports the possibility of NFAT signalling downstream of Wnt4. We therefore applied Ionomycin to cultured *Wnt4*^−/−^ kidneys to examine if the absence of nephrons could be rescued by ectopic activation of the Calcium/NFAT pathway. *Wnt4*^−/−^ kidneys cultured on standard or DMSO-supplemented media failed to undergo MET. By 72 h in culture they have no or very few renal aggregates, none of which progress beyond the comma-shaped stage (Figs. 7A, D). This phenotype was rescued when the kidneys were cultured on Ionomycin-supplemented media (Fig. 7B). The number of renal aggregates was significantly increased by Ionomycin (p = 0.047 using a two sample t-test assuming unequal variance). This effect is specific to nephrogenesis; the numbers of UB branches and tips were not increased by Ionomycin. All treated mutant rudiments developed aggregates, and both comma- and s-shaped bodies were observed (Fig. 7H). Most importantly, a distinct shift towards more mature renal MET-derived structures occurred: not only did the overall number of epithelised structures increase, but the proportion of these reaching the s-shaped state returned to that observed in wild type/heterozygous littermates (Fig. 7I). The total number of aggregates was still lower in the Ionomycin-rescued *Wnt4*^−/−^ kidneys but this is expected as *Wnt4*^−/−^ kidneys are smaller than those of wild type/heterozygous littermates at E11.5. The effect on number of s-shaped bodies is statistically significant (p = 0.033), suggesting that Ionomycin can push renal aggregates further through MET. Ectopic activation of the Calcium/NFAT pathway therefore rescues the nephrogenic defect in *Wnt4*^−/−^ kidneys, and demonstrates the sufficiency of the pathway to overcome the block in MET caused by absence of Wnt4.

## Discussion

Nephrogenesis is the process by which nephrons, the functional units of the adult kidney, are generated. It comprises a highly orchestrated series of events and intermediate forms, controlled by numerous signalling pathways. The process occurs repeatedly such that the adult human kidney contains ~ 500,000–1,000,000 nephrons, and the mouse kidney ~ 12,000 (Vainio and Lin, 2002). Low nephron number at birth is a known risk factor for renal and hypertensive problems later in life (Hoy et al., 2005). Complete failure to produce nephrons results in renal agenesis and postnatal death due to renal failure — as seen in mice with homozygous loss of *Wnt4* (Stark et al., 1994).

*Wnt4* is expressed in the condensing MM of the kidney from E11.5. Expression is then maintained in each of the intermediate epithelised structures which sequentially develop from the condensed MM during nephrogenesis: the aggregates, renal vesicles, comma- and s-shaped bodies. Down regulation occurs once the mature epithelial tubules fuse with the UB-derived collecting duct system (Stark et al., 1994). Wnt4 induces nephrogenic MET: exogenous Wnt4 is sufficient to induce MET in isolated MM (Kispert et al., 1998), whilst *Wnt4*^−/−^ mouse embryos harbour a defect in MET of the MM. *Wnt4*^−/−^ MM condenses normally but then fails to undergo complete MET, instead forming a greatly reduced number of simple renal aggregates (Stark et al., 1994). Poorly developed comma-shaped or more mature bodies may also sometimes be observed, however these are extremely rare (personal observation; (Kobayashi et al., 2005; Perantoni et al., 2005)). *Wnt4*^−/−^ mice are thus born without nephrons and consequently die of renal failure.

In this paper we have shown that components of the non-canonical Calcium/NFAT Wnt signalling pathway are present in the mid-gestation embryonic kidney. This supports previous data that a pan-NFAT antibody localises to the renal vesicles, s-shaped bodies, tubules, and UB tips at late gestation, as well as to adult tubules and a cortical collecting duct cell line (in which expression of the NFAT genes can also be detected) (Puri et al., 2004). Pharmacological suppression of the Calcineurin/NFAT pathway stunted kidney development and reduced nephron number - a phenotype similar to that in *Wnt4*^−/−^ mutants. Moreover, we demonstrate that activation of calcium signalling rescues the defect in nephrogenesis in *Wnt4*^−/−^ cultured kidneys. Not only was the overall number of epithelised structures increased in Ionomycin-treated mutant kidneys, but a significant shift towards more mature nephrogenic states was also noted. The number of comma- and s-shaped bodies increased — the latter structure never being observed in untreated mutant kidneys in our studies. Activation of calcium signalling by Ionomycin is therefore sufficient to push *Wnt4*^−/−^ kidneys through the block in MET. Due to limitations of the kidney culture system it is not possible to see whether these s-shaped bodies proceed to form fully differentiated functional nephrons; however, the rescued s-shaped bodies were morphologically comparable to those in wild type cultured kidneys, albeit smaller. The ratio of branches to aggregates however remained higher in Ionomycin-treated mutant kidneys compared with wild type kidneys, indicating that the rescue is not complete.

Previous studies of Wnt signalling in early kidney development have focused largely on the canonical β-catenin signalling pathway. In the β-catenin pathway, Wnt proteins bind to membrane-bound Frizzled receptors, activating a Dishevelled-containing complex which exerts an inhibitory effect on a second complex containing GSK3β. Once inhibited GSK3β can no longer target β-catenin for degradation and thus the stabilised β-catenin protein can translocate to the nucleus where it co-operates with a host of other factors to transactivate gene expression. The UB branches under the control of the canonical β-catenin pathway (Bridgewater et al., 2008). A number of pieces of evidence have also pointed to a pro-nephrogenic role for the canonical pathway in the MM. Removal of β-catenin activity specifically in the nephron progenitor MM inhibits renal vesicle formation (Park et al., 2007). Isolated MM cannot undergo nephrogenesis as it does not receive UB-derived inductive signals; however pharmacological activation of β-catenin signalling in isolated MM using GSK3β inhibitors partially rescues this phenotype as treated MMs initiate MET and tubulogenesis (Davies and Garrod, 1995). The induced epithelised structures do not however undergo terminal MET. In cultured kidney cells, competent to form tubules, stabilised β-catenin promotes cell growth and survival, but not tubulogenesis (Lyons et al., 2004). Similarly, adenoviral transfer of constitutively active β-catenin into isolated MM promotes cell-autonomous survival of epithelial progenitors and results in formation of renal aggregates, but not terminal MET (Schmidt-Ott et al., 2007). MM-specific genetic activation of β-catenin in cultured MM also initiates tubulogenesis, but again is not sufficient for the formation of mature epithelial structures (Park et al., 2007). *In vivo* this genetic activation can functionally replace Wnt9b and Wnt4 in the induction of nephrons — but once more is insufficient for full nephrogenic MET (Park et al., 2007).

The emergent hypothesis from these studies is that nephrogenesis may be biphasic: canonical Wnt signalling initiates MET but must then be attenuated and a second, non-canonical Wnt pathway initiated for terminal MET. In this paper we report a lack of detectable β-catenin pathway activity and the presence of components of the Calcium/NFAT pathway in the nephrogenic MM and resultant epithelised structures. Furthermore, we show that inhibition of the Calcineurin/NFAT pathway in cultured kidney rudiments disrupts renal morphogenesis and that exogenous activation of the pathway rescues the nephrogenic deficit in *Wnt4*^−/−^ kidneys. These findings support the idea that a non-canonical Wnt pathway is required for a full MET.

Can our findings be reconciled with those on the canonical pathway? It is certainly conceivable that nephrogenesis involves a number of Wnt signalling pathways, each with distinct roles at different stages, and with crosstalk between them. A biphasic program of Wnt-mediated nephrogenesis would be one possible way to marry our data with previous findings. In the first phase, from E11.5, low level activation of the β-catenin pathway in the MM would induce *Wnt4* and the initiation of nephrogenesis. Once β-catenin signalling has fulfilled its role in this initial induction it would need to be attenuated in order for complete MET to occur. Intriguingly, Wnt4 has been previously shown to inhibit canonical Wnt signalling (in HEK-293 T cells) (Bernard et al., 2008). Following attenuation, a second phase would now occur in which β-catenin signalling is down regulated and a second Wnt pathway takes over. Our data would support the role of the Calcium/NFAT pathway in this latter phase.

This biphasic model fits with previous findings: 1) that activation of β-catenin signalling is sufficient to induce nephrogenesis in isolated MM; 2) that isolated MM in which β-catenin is activated and *Wnt4*^−/−^ kidneys cannot progress beyond the comma-shaped body stage, and stabilisation of β-catenin seems to be inhibitory on MET in wild type kidneys at this stage. The model also supports the data presented here: 1) that *NFAT* genes are expressed in the developing kidney, with *NFATc1*, *NFATc2*, and *NFATc3* expression increasing as MET progresses, and NFATc3 localising to the developing nephrons; 2) that exogenous Calcium/NFAT signalling supports terminal MET in the form of s-shaped and more mature epithelial bodies in *Wnt4*^−/−^ kidneys.

A major drawback to the foregoing model is that in our studies we simply do not see activation of the β-catenin pathway during early nephrogenesis. Similarly, Wnt4 fails to activate the canonical TOPFLASH reporter in nephrogenic MM cells (Iglesias et al., 2007). In an accompanying paper Tanigawa et al., 2011 also describe that the canonical pathway is inactive in the MM. It must therefore be considered a possibility that the canonical β-catenin pathway does not endogenously induce nephrogenic MET and that the ability of pharmacologically stabilised β-catenin to induce MET may reflect its role in epithelial adherens junctions. It should also be noted that the GSK3β inhibitors used in the experiments outlined earlier are not specific to the canonical pathway. GSK3β inhibitors also prevent re-phosphorylation (and therefore nuclear export) of NFAT proteins and enhance NFATc DNA binding activity, thus facilitating activation of the Calcium/NFAT pathway (Beals et al., 1997; Neal and Clipstone, 2001). Tanigawa et al., 2011 have now generated a peptide which specifically targets canonical Wnt signalling. Using this peptide they were able to show that disruption of canonical Wnt signalling does not disrupt tubule formation. Furthermore they demonstrate that Wnt4 induces Ca^2+^ influx, CaMKII phosphorylation, and activation of an NFAT reporter construct in MM cells, and that Ionomycin promotes nephrogenesis in isolated MM. This pro-nephrogenic effect complements our finding that nephrogenic genes are up-regulated in Ionomycin-treated kidneys, particularly *Six2*. Nephrogenesis may therefore initiate solely due to non-canonical Wnt signalling *in vivo*. The fact that the overall number of renal aggregates in Ionomycin-treated *Wnt4*^−/−^ kidneys increased would support this model. We also occasionally noted aggregates forming ectopically (away from UB tips) in the MM of wild type kidneys treated with Ionomycin (Fig. 5B), again supporting a pro-nephrogenic effect of calcium signalling.

Alternatively, a biphasic system could still exist if the UB were the source of the canonical Wnt signal. It is plausible that canonical signalling from the UB, possibly activated by Wnt9b (Carroll et al., 2005), induces *Wnt4* expression in the MM and that the canonical pathway can act similarly when ectopically activated in the MM. A final possibility is that canonical β-catenin signalling has a role in nephrogenesis at stages later than those on which we have focused. Indeed, Mugford et al., 2009 detected activation of an Axin2-LacZ knock-in construct in the kidney at E15.5 but not E11.5. We did not see this activity in our BAT-gal mice but differences in activity between transgenes reporting on different components of a pathway are to be expected.

A final issue to address is the lack of kidney abnormalities in NFATc gene mutants. Mice homozygous for loss of *NFAT5* display renal atrophy (Lopez-Rodriguez et al., 2004), but the product of this gene is not calcium-regulated. Of the knockout models generated for the four *NFATc* genes, none display obvious renal defects (personal communications). However, the major cardiac and brain developmental defects associated with loss of these genes only become apparent in double and triple knockout mice, indicating that functional redundancy exists between NFATc gene family members (Arron et al., 2006). It is therefore interesting to note that homozygous loss of the gene encoding the Calcineurin A-α catalytic subunit (*Ppp3ca*) results in a reduced nephrogenic zone and renal agenesis (Gooch et al., 2004). Moreover, *Ppp3ca* is strongly expressed in the cap MM where nephrogenesis occurs and *Wnt4* is expressed (Andrew McMahon lab data, deposited in the renal expression database at http://www.gudmap.org). As Calcineurin regulates all four of the NFATc transcription factors its loss would therefore abrogate all downstream NFATc activity and bypass redundancy between the NFAT proteins. We therefore propose the non-canonical Wnt Calcium/NFAT pathway as a novel regulator of nephrogenesis.

## Materials and methods

### Kidney organ culture

T-bud stage kidneys were isolated from E11.5 mouse embryos and cultured at 37 °C with 5% CO_2_ on 0.4 μm pore Transwell filters (Costar 3450) suspended on Minimum Essential Medium Eagle medium (Sigma M5650) supplemented with 10% foetal calf serum and 1% Penicillin/Streptomycin. CSA, FK506, Ionomycin, ethanol, and DMSO were added to the media underlying the filter where required at the concentrations stated in the text/figures.

### Immunohistochemistry, X-gal staining, and *in situ* hybridisation

Immunohistochemistry was performed on cultured or microtome-sectioned kidneys. Cultured kidneys were fixed in ice-cold methanol for 10 min, washed in phosphate buffered saline (PBS), blocked overnight in PBS/2% BSA/0.01% sodium azide at 4 °C, incubated overnight at 4 °C with primary antibodies in PBS/BSA/azide, washed in PBST (1% Triton), incubated overnight with secondary antibodies in PBS/BSA/azide at 4 °C, washed in PBST, and mounted in Vectashield (Vectorlabs).

For section immunohistochemistry, whole embryonic kidneys were fixed in 4% paraformaldehyde (PFA), washed in PBS, dehydrated through an ethanol series, cleared using Histoclear (National Diagnostics), embedded in paraffin wax, and sectioned using a microtome. Sections were dewaxed in Xylene, washed in ethanol and, if they were to undergo immunoperoxidase antibody detection, blocked for endogenous peroxidase activity with 0.3% hydrogen peroxide. Following dehydration in 70% ethanol, sections were washed in water, subjected to microwave antigen retrieval in Tris/EGTA buffer, washed in 50 mM ammonium chloride, washed in PBS containing 0.2% gelatin/1% BSA/0.05% Saponin, and incubated with primary antibodies at 4 °C overnight. The next day sections were washed in 0.2% gelatin/0.1% BSA/0.05% Saponin in PBS prior to incubation with secondary antibodies at room temperature. Incubation with peroxidase-conjugated secondary antibodies was followed by washes in 0.2% gelatin/0.1% BSA/0.05% Saponin, DAB treatment, PBS and water washes, counterstaining with Haematoxylin, dehydration, clearing in Xylene, then mounting in DePeX. Sections incubated with fluorescent secondary antibodies were washed in PBS and water then mounted using Vectashield.

Primary antibodies used were as follows: anti-calbindin (D28K, clone CL-300, ab9481, Abcam; D28K, clone C-20, sc-7691, Santa Cruz), anti-Pax2 (71–6000, Invitrogen), anti-dephosphorylated β-catenin (clone 8E4, C-1202, AG Scientific), anti-pan β-catenin (C2206, Sigma; 95825, Cell Signaling Technology) , anti-NFATc3 (F-1, sc-8405, Santa Cruz), and anti-laminin (L9393, Sigma).

Whole kidneys were fixed with 4% PFA/PBS prior to overnight X-gal staining at 37 °C. Stained fixed kidneys were embedded and sectioned as before, and sections counterstained with Nuclear Fast Red (Vector Laboratories). Cultured kidneys were fixed in 4% PFA/PBS then X-gal stained overnight at 37 °C and re-fixed.

*NFATc3* and *NFATc4* expression was analysed by *in situ* hybridisation, using DIG-labelled antisense RNA probes reverse transcribed from a PCR product template (NFATc3 F: CCTTTGAGTGCCCAAGTATTC, R: CGATGTTAATACGACTCACTATAGGGCTGAGGCCTGATCCAGTGTGG; NFATc4 F: ATAGCTGGCTACTCCTCAGC, R: CGATGTTAATACGACTCACTATAGGGCACCATCTTGCCAGTAATCCG). *In situ* hybridisation was performed essentially as described by Wilkinson (1992). Detection was performed with BM Purple (Roche Applied Science).

### Mice

All animal experiments were approved by the University of Edinburgh ethical committee (UK) or conducted under PHS guidelines and approved by the relevant Institutional Animal Care and Use Committees (USA). Experiments were conducted on CD1 wild type mice, unless otherwise stated. BAT-gal, Wt1-GFP, and TOPgal transgenic mice have been previously described (DasGupta and Fuchs, 1999; Hosen et al., 2007; Maretto et al., 2003). The TCF/Lef:H2B-GFP transgene comprises six copies of a Lef/TCF binding site, upstream of an Hsp68 minimal promoter and an H2B-GFP fusion reporter. A detailed characterisation of the TCF/Lef:H2B-GFP Wnt reporter line will be provided elsewhere (Ferrer-Vaquer et al., 2010). Embryonic day 0.5 (E0.5) was the morning when a vaginal plug was detected.

### Real-time quantitative RT-PCR

RNA was isolated from whole wild type E11.5–15.5 embryonic kidneys using an RNeasy Mini kit (QIAGEN). For RNA isolation after drug treatment kidney rudiments were stored in RNAlater (Ambion), subsequently lysed in RLT buffer with β-mercapto-ethanol on the filter, and further treated as per manufacturer's instructions using the same kit. Control samples were treated with solvent only (EtOH for FK506 and CSA; DMSO for Ionomycin). GFP-positive cells were isolated from E12.5 *Wt1-GFP* transgenic kidneys using a FACSaria II cell sorter (BD) and RNA isolated from GFP-sorted cells with an RNeasy Micro kit (QIAGEN). cDNA was subsequently synthesised using a First Strand cDNA Synthesis Kit (Roche). Taqman RT-PCR using the Universal Probe Library (Roche) was performed according to the manufacturer's instructions as follows: NFATc1 (NFAT2) — F: tccaaagtcattttcgtgga, R: tttgcttccatctcccagac, probe 50; NFATc2 (NFAT1) — F: gatcgtaggcaacaccaagg, R: cttcaggatgcctgcaca, probe 1; NFATc3 (NFAT4) — F: caagatggaagacctcattgg, R: gggaggaacttcaaggacaa, probe 41; NFATc4 (NFAT3) — F: cggcttcagagacagtgtacc, R: gtctcggccaatgatctcac, probe 69; NFAT5 — F: gacaccttcttcccccattt, R: gcttttctttaagatctcaggaactc, probe 102; Ret — F: tggagtttaagcggaaggag, R: acatctgcatcgaacacctg, probe 49; Wnt11 — F: gagctcgcccccaactac, R: ggcatacacgaaggctgact, probe 85; Ksp-cadherin — F: ggttgtccaccatgataccc, R: tgcagcgacacacaatcac, probe 85; Six2 — F: caagtcagcaactggttcaaga, R: actgccattgagcgagga, probe 5; CD15 — F: tggtactacgcgtgttcgac, R: ccagggctttgccagtta, probe 32; Synaptopodin — F: ggaaagtgatgacagccagtg, R: ttttcggtgaagcttgtgct, probe 104. All assays used a commercially available UPL compatible Gapdh assay (Roche) as internal control. All assays except for CD15 (which is encoded by the single exon *Fut4* gene) were intron-spanning.

The following are the supplementary materials related to this article.
